# Hand grip strength: Reference values for adults and elderly people of Rio Branco, Acre, Brazil

**DOI:** 10.1371/journal.pone.0211452

**Published:** 2019-01-31

**Authors:** Cledir Araújo Amaral, Thatiana Lameira Maciel Amaral, Gina Torres Rego Monteiro, Mauricio Teixeira Leite Vasconcellos, Margareth Crisóstomo Portela

**Affiliations:** 1 Federal Institute of Acre, Rio Branco, Acre, Brazil; 2 Center for Health Sciences and Sports, Federal University of Acre, Rio Branco, Acre, Brazil; 3 Department of Epidemiological and Quantitative Methods in Health, National School of Public Health Sergio Arouca, Oswaldo Cruz Foundation, Rio de Janeiro, Rio de Janeiro, Brazil; 4 National School of Statistical Sciences, Brazilian Institute of Geography and Statistics, Rio de Janeiro, Rio de Janeiro, Brazil; 5 Department of Health Administration and Planning, National School of Public Health Sergio Arouca, Oswaldo Cruz Foundation, Rio de Janeiro, Rio de Janeiro, Brazil; Federal University of Pelotas, BRAZIL

## Abstract

Hand grip strength (HGS) is recognized as an important health indicator, but validated reference values that can be applied to the evaluation of individuals in different populations are still lacking. This work aimed to identify correlations between HGS and anthropometric variables and to establish HGS reference values for adult and elderly populations. This is a population-based cross-sectional study considering the subsets of individuals with healthy right or left upper limbs from a sample of 1,609 adults and elderly residents in Rio Branco, Acre, Brazil. Descriptive statistics of anthropometric measures and HGS values at maximum performance based on three measurements of the two hands were obtained, and Pearson correlations between these variables were applied. Percentile distributions were estimated for right and left HGS by sex and age group. Men presented, in general, a maximum HGS 57% higher than women (43.4 kg vs. 27.6 kg), and also higher HGS levels in the different age groups. In both sexes, the highest HGS values were observed in the age group of 30 to 39 years (men, 46.9 kg; women, 29.4 kg), with a subsequent decline. HGS presented a negative correlation with age and a weak to moderate positive correlation with anthropometric variables, among men and women. The median HGS of men was reduced by about 46% between the ages of 30 and 39 years and 80 years and over (right hand, 46.4 to 23.7 kg; left hand, 42.2 to 23.5 kg) and by about 44% in women (right hand, 29.0 to 16.4 kg, left hand, 27.3 to 15.2 kg). The values identified are a reference for HGS behavior among healthy adults and seniors, although they do not discriminate individuals with specific health conditions. They can be used in rehabilitation programs and subsidize future studies aimed at exploring their potential application in the evaluation of the health condition of adults and elderly individuals.

## Introduction

Hand grip strength (HGS) is used clinically in the rehabilitation area [1,2] and has been recommended as a basic measure in the determination of musculoskeletal function, as well as of weakness and disability [3–5]. The HGS, measured by manual dynamometry, produces a measure of isometric strength that allows identifying not only muscle weakness of the upper limb, but also providing an indicative of overall strength, since it reflects the strength of the lower limbs [6]. This gives it an important role in the evaluation of functionality [1]. Manual dynamometry is a relatively simple, fast, inexpensive and non-invasive test; thus HGS is considered a good health marker [7].

HGS is influenced by age, sex, anthropometric variables (height, weight, hand size, arm circumference) and hand dominance [8–11], and is associated with different health outcomes [9,12], especially in elderly people [7]. It plays an important role in the evaluation of clinical and surgical treatment prognoses [13–15], functional evaluation of the elderly [16], identification of potential sports talents [17] and in the composition of the battery of admission tests in different professional activities such as police, armed forces and fire brigade [18]. Thus, the availability of reliable and up-to-date population reference values to which individuals can be compared is paramount [19].

There is a growing number of studies reporting HGS reference values [9,20,21], but their generalization is hampered by the variability of measurement instruments and protocols [22,23], differences between baseline populations, and use of non-representative samples [8,16]. Brazilian studies published on reference values for the population [8,24,25] are neither comparable nor generalizable to the entire Brazilian population, which presents diverse physical, cultural, social and economic variation from region to region. It is possible to observe very different HGS patterns from one region to another in the country.

The establishment of HGS reference values for different populations makes it possible to detect differences between them but also serves to subsidizing efforts to construct more comprehensive or generalizable reference values. The understanding of the behavior of HGS in the population is important to create parameters in physical rehabilitation programs, as well as for the exploration of HGS levels discriminating the risk of occurrence of health conditions. This study aims to identify correlations between HGS and anthropometric variables and to establish HGS reference values for adult and elderly population groups based on data collected by the Study of Chronic Diseases (*Estudo das Doenças Crônicas—EDOC*), conducted in Rio Branco, Acre, Brazil.

## Material and methods

The EDOC is composed of two domiciliary surveys conducted between April and September 2014: EDOC-A with adults (18–59 years) and EDOC-I with the elderly (60 years and over) residing in Rio Branco, AC. Pregnant women and individuals with cognitive impairments that could prevent communication or the understanding of the questions were excluded from the research population. Sampling plans were selected in two stages, census enumeration area (CEA) and domicile, being the first stage common to both surveys. The selection of the CEAs was made with a probability proportional to their number and private households in the IBGE Demographic Census 2010 (CD2010). Households were selected by systematic sampling with random starts and distinct intervals by survey. In the households selected for the EDOC-A, all adult residents were interviewed, and in the households selected for the EDOC-I, all the elderly were interviewed.

The sample size was calculated considering the prevalence of renal impairment of 15% in adults and 40% in the elderly [26,27], with 95% confidence level and 3% absolute error [28] for simple random sampling of proportions. Considering that the sampling plan is a conglomerate per CEA, a sampling plan effect of 1.95% was used to determine the sample sizes, with an additional of 20% for the EDOC-A and 12.5% for the EDOC-I to compensate for the expected missing responses. This procedure resulted in samples of 652 adults and 1,148 elderly. Dividing these sample sizes by the average number of adults and elderly people per household obtained in demographic census DC2010 and defining the selection of 11 households per CEA for the EDOC-A and 73 households for the EDOC-I, a size of 40 households was obtained for the sample of CEAs. The effective sample consisted of 685 adults and 1,016 elderly people interviewed.

Sample weights were calculated inversely to the probabilities of inclusion at each stage and were later calibrated to population data according to sex and age groups using a post-stratification estimator in order to deal with the typical biases of household surveys and to correct for differential missing responses [29]. The population data used for calibration of sample weights were estimated for July 1, 2014, using the linear trend method [30] that the IBGE applies in its population estimates per municipality. However, 42 adults and 50 elderly people of the original samples did not have HGS measured, which generated subsamples that had their sample weights corrected and recalibrated to produce estimates for 211,902 adults and 23,416 elderly. Further details on the EDOC sampling plan, calculation and calibration of sample weights and subsamples was showed by Amaral and cols. [31].

The surveys involved home interviews based on a structured questionnaire that included socioeconomic, demographic and health information.

As described by Amaral e cols. [31] all data collection procedures were performed by qualified personnel, trained and supervised by the coordination of the study. The team of interviewers was composed by students and health professionals who participated in a preparatory course promoted by the coordination to understand the role, function and importance of the interviewer in scientific research, in addition to the familiarity, understanding and application of the instruments. To standardize the interviews, a manual for study and activities basis was prepared. The team responsible for the physical assessment, vital signs and collection of biological samples, named the health team, was composed of health professionals: nurses, physical education professional, nursing and laboratory technicians. All professionals were previously trained in order to understand and standardize the procedures adopted.

Anthropometric evaluations were performed, considering the mean of two measurements collected for weight, height and right arm, waist, hip and right calf circumferences, according to measurement protocols recommended by the American College of Sports Medicine [32]. With participants wearing light clothes and without accessories, barefooted, erect body and weight equally distributed between the two feet, arms at the side of the body, and looking straight ahead, the height was determined by a Sanny^®^ mobile stadiometer, with resolution in millimeters and base placed on a flat surface. The weight was measured through a G-Tech^®^ Bal Gl 200 digital scale, with a resolution of 50 grams, laid out on a flat surface.

The circumference measurements, in centimeters, were made with a Cescorf^®^ inelastic anthropometric tape, with resolution in millimeters, positioned in the body regions in an adjusted manner, without crinkling the skin or compressing the cutaneous tissues, with reading on the side of the assessed individuals. In order to obtain the arm circumference, the tape was placed on the midpoint of the right arm, parallel to the ground; and to waist circumference, on the midpoint between the iliac crest and the first rib, with participants breathing normally, relaxed abdomen, and reading of the measurement at the end of the expiration. The measurement of the hip circumference was made with the tape positioned at the largest circumference of the buttocks in the horizontal plane, with participants with arms slightly distanced at the front and with feet together. The calf was measured with the individual in the orthostatic position, feet slightly distanced, and body weight equally distributed, the tape placed in the portion with largest circumference [32].

The body mass index (BMI) was defined as the weight (kg) divided by the square of height (m^2^).

HGS was expressed in kg and measured using a hydraulic hand dynamometer (SAEHAN SH5001^®^), previously calibrated, with a resolution of 2 kg, following the procedures adopted by the American Society of Hand Therapists [33]. Measurements were obtained in standardized conditions, with the participants in the seated position, elbow at 90°, handle adjusted to the second position and after receiving explanation on the procedures and after familiarizing with the instrument, they should apply the maximum grip strength for 3 to 5 seconds. The procedure was performed three times with each hand alternately, with an interval of one minute between each measurement.

In the analyses presented here, all individuals with both upper limbs defined in the study as not healthy due to reports of previous injury, recent acute pain or malformation that could affect the performance in the HGS test were excluded. Two subsets were defined taken from the set of 1,462 individuals with at least one healthy upper limb, corresponding to 217,477 individuals in the population, one subset with 1,310 observations on the right hand, and another with 1,358 observations on the left hand, allowing inferences about the right and left HGS of 193,894 and 206,124 people in the population, with the right and left upper limbs considered healthy, respectively.

For the analyses of this study, the highest HGS value of three observations for each hand was considered. The variables "HGS R" and "HGS L", were created to designate, respectively, the maximum strength of the right and left hand. Additionally, the variable "Max HGS" was created to express the largest value between "HGS R" and "HGS L", or the only one existing, in case one of the upper limbs was not healthy. All data is available in S1 Dataset.

Descriptive statistics were obtained focusing on measures of central tendency and dispersion (mean, median, minimum value, maximum value, standard error and coefficient of variation), identifying the number of observations of the sample (n) and population estimates (N), in addition of the percentile distribution of HGS of the right and left hands stratified by sex and age group (18–29, 30–39, 40–49, 50–59, 60–69, 70–79 and 80 and over). Pearson correlation coefficients between the right and left HGS values and the variables age, weight, height, BMI and arm, waist, hip and calf circumferences were estimated, considering the significance level of 5%. In all the analyses, the effect of the sample design (complex sample) and the weights of the observations were taken into account using the "*proc survey*" procedures of the SAS^®^ statistical package version 9.4.

The survey was approved by the Research Ethics Committee (REC) of the Federal University of Acre under CAAE: 17543013.0.0000.5010, all participants signed an Informed Consent Term. This study, specifically, was also approved by the REC of the National School of Public Health Sérgio Arouca, CAAE 50895015.2.0000.5240.

## Results

Among the population of adults and elderly people with at least one healthy upper limb, 37.9% were men and 62.1% were women, and the age ranged from 18 to 102 years, with an average of 37.6 (± 0.5) and a median of 33.1 years.

Males presented mean and median greater HGS values and anthropometric measurements than those observed in women, except for BMI and hip circumference (Table 1). Mean values of right and left HGS were, respectively, 42.4 and 40.6 kg in men, and 27.1 and 25.9 kg in women. In both men and women, right HGS was greater than the left HGS, and the left lateral dominance was 8.0% in the population.

**Table 1:** Measures of central tendency and dispersion of right and left HGS (kg), age and anthropometric variables according to sex in adults and elderly individuals in Rio Branco-AC. Brazil, 2014.

Variables	Sample			Population Estimates			
	n	Min	Max	Mean	SD	Median	CV
**Men**							
HGS R	496	10.0	74.0	42.6	10.17	42.2	0.239
HGS L	516	6.0	67.0	40.9	9.58	40.1	0.234
Age	554	18	102	37.6	14.80	32.7	0.394
Weight (kg)	554	34.4	133.7	75.3	16.70	73.0	0,222
Height (cm)	548	1.42	1.88	1.69	0.08	1.69	0.047
BMI (kg/m^2^)	548	13.4	46.8	26.2	5.20	25.5	0.198
AC (cm)	550	13.0	46.0	31.3	4.78	30.9	0.153
WC (cm)	552	53.0	138.0	87.0	12.78	85.9	0.147
HC (cm)	550	53.0	148.0	97.7	9.28	96.8	0.095
CC (cm)	548	18.0	48.0	35.5	4.09	35.8	0.115
**Women**							
HGS R	814	6.0	51.0	27.1	6.32	26.0	0.233
HGS L	842	3.0	54.0	25.8	6.36	24.7	0.247
Age	908	18	109	37.6	14.72	33.3	0.391
Weight (kg)	907	25.8	120.0	66.1	13.50	64.6	0.204
Height (cm)	902	1.29	1.95	1.58	0.07	1.57	0.044
BMI (kg/m2)	901	12.5	51.1	26.7	5.16	26.1	0.193
AC (cm)	906	10.0	47.0	29.9	4.91	29.9	0.164
WC (cm)	907	45.0	138.3	81.3	12.14	80.0	0.149
HC (cm)	905	51.0	150.0	99.4	10.73	98.8	0.108
CC (cm)	900	14.0	81.0	34.5	4.12	34.5	0.119

HGS R = right hand grip strength; HGS L = left hand grip strength; BMI = body mass index; AC = arm circumference; WC = waist circumference; HC = hip circumference; CC = calf circumference; n = sample size; Min = minimum; Max = maximum SD = Standard Deviation (Estimated by Taylor Series Linearization Method); CV = coefficient of variation.

Despite the lower and upper limits of the measurements obtained, the distributions of HGS values and anthropometric variables in the population, based on the coefficients of variation estimated, are not so heterogeneous.

The mean maximum HGS observed among men (43.4 kg) was about 57% higher than that observed among women (27.6 kg) and, for both sexes, it reached the apex in the age range of 30 to 39 years—46.9 kg and 29.4 kg, respectively, in men and women (Table 2).

**Table 2:** Mean and standard deviation of maximum Handgrip Strength (HGS) according to sex and age in the population of Rio Branco-AC. Brazil, 2014.

	Total			Men			Women		
Age (years)	n	Mean	SD	n	Mean	SD	n	Mean	SD
18–29	183	36.2	11.57	46	44.7	10.56	137	28.6	6.29
30–39	136	38.0	12.05	48	46.9	10.39	88	29.4	6.39
40–49	131	35.1	10.05	40	42.7	8.79	91	28.3	5.66
50–59	143	32.7	10.97	50	41.2	8.65	93	24.2	6.06
60–69	415	29.4	9.27	169	36.2	8.15	246	23.0	5.55
70–79	298	25.5	7.95	131	31.3	6.97	167	20.3	5.05
80 and over	156	21.1	6.78	70	25.7	5.81	86	17.1	4.98
**Total**	**1,462**	**35.2**	**11.55**	**554**	**43.4**	**10.50**	**908**	**27.6**	**6.58**

n = sample size; Mean = mean of the highest HGS value among three measurements of each hand whose upper limb was classified as healthy; SD = Standard Deviation (Estimated by Taylor Series Linearization Method).

From the age of 40 onwards, there was a progressive decline in HGS, which was more evident in the 50–59 age group in the case of women, and 60–69 years in men. From the age of 60 onwards, men had a relative greater loss than women.

In both sexes, a strong correlation was observed in the crossing of right and left hand dynamometry. In men and women, negative, statistically significant correlations of right and left HGS with age were found. Among men, right and left HGS were significantly and positively correlated with height, weight, and arm, hip and calf circumferences; BMI and waist circumference were positively correlated only with left HGS. Among females, statistically significant positive correlations of HGS in both hands were observed with height, weight, BMI and arm, hip and calf circumferences. It is worth emphasizing, however, that, except for the correlations between right HGS and left HGS with height and the right HGS with arm circumference among men, which were moderate, all correlations of HGS with anthropometric measurements were considered weak or very weak (Table 3).

**Table 3:** Correlations between right and left HGS and age and anthropometric variables according to sex in the population of Rio Branco-AC. Brazil, 2014.

	HGS R	Age	Height	Weight	BMI	AC	WC	HC	CC
**Men**									
HGS R	1	-0.261*	0.524*	0.371**	0.163	0.431*	0.154	0.269**	0.308**
HGS L	0.867*	-0.284*	0.508*	0.340*	0.140**	0.369*	0.134**	0.237**	0.311*
**Women**									
HGS R	1	-0.300*	0.324*	0.291*	0.160*	0.220*	0.086	0.175*	0.320*
HGS L	0.854*	-0.321*	0.331*	0.294*	0.147**	0.221*	0.060	0.179**	0.367*

HGS R = right hand grip strength; HGS L = left hand grip strength; BMI = Body Mass Index; AC = circumference of the right arm; WC = waist circumference; HC = hip circumference; CC = circumference of the right calf.

* p-value < 0.001;

** p-value < 0.05.

The percentile distribution analysis of HGS showed an increase in strength up to the age range of 30 to 39 years, followed by a progressive decline with advancing of age in both hands, in both men and women. Among the age groups considered, the median values of right and left HGS ranged from 46.4 to 23.7 kg and 42.2 to 23.5 kg among men, and from 29.0 to 16.4 kg and 27.3 to 15.2 kg among women (Tables 4 and 5).

**Table 4:** Percentile distribution of right and left HGS according to age group in men from Rio Branco-AC. Brazil, 2014.

	18–29 years	30–39 years	40–49 years	50–59 years	60–69 years	70–79 years	≥ 80 years
**Percentile**	**Right hand grip strength (kg)**						
**05**	19.3	23.8	19.8	20.4	17.9	19.3	12.3
**10**	24.3	30.4	27.1	23.7	21.5	20.4	15.0
**25**	35.1	37.9	38.4	36.9	29.8	25.2	19.7
**50**	**42.9**	**46.4**	**42.1**	**42.1**	**35.3**	**30.0**	**23.7**
**75**	51.5	50.5	51.6	45.9	39.7	35.8	28.6
**90**	55.6	60.0	54.4	49.5	45.7	39.7	33.0
**95**	57.5	64.0	55.4	50.4	48.6	43.0	35.9
	**Left hand grip strength (kg)**						
**05**	21.1	25.3	25.0	17.7	20.5	18.4	11.0
**10**	26.9	29.8	27.0	22.7	22.9	19.7	16.2
**25**	34.8	37.9	35.4	30.7	28.6	23.9	18.7
**50**	**42.2**	**42.0**	**39.3**	**38.2**	**34.9**	**28.5**	**23.5**
**75**	48.4	51.0	46.5	44.1	38.8	33.3	28.6
**90**	53.9	53.9	49.6	48.2	44.2	38.6	32.0
**95**	55.3	62.4	52.7	49.8	47.4	40.6	33.7

**Table 5:** Percentile distribution of right and left HGS according to age group in women from Rio Branco-AC. Brazil, 2014.

	18–29 years	30–39 years	40–49 years	50–59 years	60–69 years	70–79 years	≥ 80 years
**Percentile**	**Right hand grip strength (kg)**						
**05**	19.0	18.0	19.4	14.3	12.0	10.2	7.9
**10**	19.7	20.6	20.3	16.8	15.7	13.2	9.8
**25**	22.1	24.1	23.0	20.3	19.0	16.6	11.7
**50**	**26.3**	**29.0**	**26.6**	**23.0**	**21.1**	**19.5**	**16.4**
**75**	31.2	33.0	29.5	28.3	25.6	22.4	19.7
**90**	36.5	37.1	33.3	30.5	28.8	26.5	21.9
**95**	39.5	39.2	37.2	31.9	31.7	28.7	25.4
	**Left hand grip strength (kg)**						
**05**	17.4	15.2	16.1	12.3	12.1	9.7	6.0
**10**	18.7	19.3	18.4	15.3	14.0	11.4	7.6
**25**	21.4	21.8	21.6	18.8	17.7	14.5	11.3
**50**	**25.3**	**27.3**	**25.7**	**21.6**	**20.4**	**17.8**	**15.2**
**75**	29.9	30.9	29.7	25.4	24.5	21.4	18.1
**90**	35.0	34.4	33.7	28.6	28.0	26.3	20.3
**95**	38.1	37.1	34.6	31.2	29.2	29.1	26.0

Among men, the reduction of the mean right and left HGS observed at each decade after reaching the peak (30 to 39 years), varied between 4.3 and 22.7 kg and 2.7 and 18.5 kg. The relative reduction between the ages 30 to 39 years and 80 years and over was 48.9% and 44.0% in the right and left hand (Table 4).

Women, on the other hand, presented a reduction of HGS in each decade since the age 30 and 39 years onwards varying between 2.4 and 12.6 kg and 1.6 and 12.1 kg for the right and left hand. The relative reduction of HGS in the right and left hands since the apex up to 80 years and over was 44.3 and 44.4% (Table 5).

## Discussion

This is, to our knowledge, the first study to present HGS reference values for the general population above 18 years of age in the Brazilian Amazon region, with the advantage of the possibility of extrapolation of these results to the adult and elderly population residing in the capital of Acre, because statistical techniques were used to sampling population data from household surveys.

HGS reference values were identified, ratifying the maximum performance of the variable in the fourth decade of life, the tendency of reduction with advancing age after reaching the maximum performance and the fact that the men are stronger than women [20,24,34,35]. Differences were observed in the typical values found and not, necessarily, in the behavior of HGS in the different age groups.

A systematic review with HGS reference values in different countries [20] found that the magnitude of HGS relates to the level of development of the country. For example, men from developed regions had a mean HGS of 52.8 kg, while in developing countries, men had a HGS of 43.4 kg, similar to that value found in the present study.

The first Brazilian study to provide HGS reference values had a convenience sample of individuals aged 20 to 59 years with healthy upper limbs and found the highest averages of 46.3 and 42.7 kg in men aged 25 to 29 years, and 32.9 and 29.6 kg in women aged 35 to 39 years, for dominant and non-dominant hands [25]. In another study carried out in Pelotas, the highest mean HGS values were 43.4 and 40.4 kg in men aged 18 to 30 years and 24.0 and 20.9 kg in women aged 31 to 59 years, considering the dominant and non-dominant hands, respectively [8]. Although the present work used the same measurement technique and the hydraulic dynamometer of the last study, the results presented here are not comparable due to the option of considering the mean of the measurements and grouping the larger age groups (18 to 30 years, 31 to 59 years and 60 years and over). Moreover, none of the two studies mentioned allow extrapolations for the general population.

A population-based study conducted in Rio de Janeiro also found that the highest mean of HGS occurs between 30 and 39 years in both sexes, being 46.5 and 44.5 kg in men, and 28.0 and 26.7 kg in women, in the right and left hands, respectively [24]. Despite the differences in technique and measurement instrument employed, these results are close to those presented here, suggesting that the estimation of HGS in the Brazilian population in general, coming from population bases, may be little affected by the choice of measuring techniques and/or instruments, or even regional characteristics. Further population studies are needed in different regions of the country to consolidate a base of information that results in national HGS reference values.

In a previous population study carried out in Rio Branco that analyzed the association of HGS with referred morbidities, the mean peak of HGS also occurred in both sexes up to 39 years of age [12], with maximum HGS values close to those reported here.

The analysis of the mean decline in HGS per decade after the age of 40 revealed that men have a greater proportional loss of strength than women in all age groups except 50–59 years. This finding is in agreement with a longitudinal study in which the HGS peak was found at 36 years of age in both sexes, with the most expressive decline starting first among women, around the age of 50, and a little later among men, from the age of 60 years onwards [36]. From the age of 60, men have higher proportional HGS loss than women [37].

HGS is directly related to muscle mass and this is reflected in the differences of strength observed between the sexes; men, in general, have a greater muscle mass than women. Hormonal differences may explain higher levels of muscle mass and strength in men [38] and are strongly affected by the process of sexual maturation, which occurs earlier among women. The behavioral influence determined especially by culture is also undeniable. This influence establishes a distinction between physical and labor activities of men and women since childhood [39]. These biological and cultural elements explain at least partially the differences in both the maximum HGS levels and their earlier reduction in females.

Although recognizing that in general the pattern of variation of HGS at the onset of life occurs similarly in the sexes, it should be noted that possibly due to lower levels of strength than those observed among men, older women tend to suffer the consequences of loss of strength in a more drastic manner, as observed in a study that identified a higher risk of all-cause mortality in this group [40]. On the other hand, strength loss was associated with a higher risk of mortality or occurrence of cardiovascular events among middle-aged men [41]. The reference values presented herein may be useful for the early detection of low HGS, allowing the implementation of resistance training, which seems promising to prevent the adverse effects of low strength in both sexes.

In studies of HGS, it is necessary to take into account the differences between the sexes and age groups, but it is also important to recognize its relation with anthropometric variables indicative of nutritional status [42]. In this study, both men and women showed negative correlations of HGS with age and positive correlations with weight, height, BMI, and arm, hip and calf circumference. These findings are corroborated by a study carried out in the United Kingdom, analyzing the HGS of people aged 39–73 years that confirmed the superiority of HGS in men in relation to women and identified an inverse correlation of HGS with age (-0.18, right hand; and -0.18, left hand, p < 0.01) and a positive correlation of HGS with height (0.67, right hand; and 0.66, left hand, p < 0.01) [34]. They are also endorsed by a recent study with Portuguese elderly that identified negative correlations of HGS with age (-0.44 and -0.42 for women and men, p < 0.001) and positive correlations with height (0.34 and 0.40, p < 0.001), arm circumference (0.19 for males, p < 0.001) and calf circumference (0.19 for females, p < 0.001). The latter study also showed a correlation between HGS and nutritional status measured by a questionnaire (0.19 and 0.16 for women and men, p < 0.001) [43], supporting the usefulness of HGS to assess changes in nutritional status [2,13,44].

It is worth noting, however, that since the correlations observed in the present study were moderate or weak, the stratification of percentile values of HGS by categories of anthropometric variables, such as height or BMI, as adopted in previous studies, was not considered necessary [34,45]. The choice of stratification of HGS only by sex and age group facilitates its use as a reference in health evaluations [24].

Additionally, the study did not account for the role played by physical activity and diet on HGS, despite the recognition of their importance in exploratory or prospective evaluations involving HGS. There is evidence that mid age recreation and occupational physical activity [46], as well as healthy diet [47], affect positively HGS levels in the elderly.

The option for presenting reference values according to laterality (left and right hand) and not to hand dominance (dominant and non-dominant) was justified by the fact that only 8% of the respondents had the left-handed. The literature indicates that only 10% of right-handed people have higher HGS in the left hand and among the left-handed there is no statistically significant difference between right and left HGS [23].

Although out of the scope of this study, it should be emphasized that about 10% of men and women aged 18–29, men aged 40–59, as well as women in the 50–59 age group could be classified as having the phenotype of sarcopenia based on the HGS cut-off points established by the European Consensus (30 kg for men and 20 kg for women) [48]. The proportion of elderly people of both sexes who would be classified as presenting sarcopenia at age 60 exceeds 25%, at age 70, 50%, and more than 75% in the population aged 80 and over. This reinforces the need for studies on HGS and sarcopenia.

We recognize as a limitation of this study the non-discrimination of healthy people from those with chronic or acute health conditions potentially related to HGS. However, to minimize this limitation, we considered only data from people with no current or previous problems in the upper limbs, including the shoulder girdle and cervical region, which might impair performance in the HGS test. HGS values presented herein as a reference may be employed in the clinical habilitation of upper limb function. They also serve as a global assessment component for adults and elderly individuals, especially in primary care, providing criteria for early identification of subjects with strength below the expected. Such subjects could be sent to a more specific evaluation to identify health problems and prevent future limitations and disabilities.

We believe that the results presented here can serve as a basis for proposing generalizable reference values at the national level.

## Conclusion

The values identified are a reference for HGS behavior among adults and seniors with healthy upper limbs, although they do not discriminate individuals with specific health conditions. They can be used in rehabilitation programs and subsidize future studies aimed at exploring their potential application in the evaluation of the health condition of adults and elderly individuals.

Further studies that may contribute to the understanding of the relationship between HGS and morbidity and mortality in adults and elderly people, as well as the quality of life, physical activity and functional autonomy, are therefore recommended.

## Supporting information

S1 Dataset Dataset. (RAR)

## References

[1] Bohannon RW. Reference values for extremity muscle strength obtained by hand-held dynamometry from adults aged 20 to 79 years. Arch Phys Med Rehabil. 1997 1 1;78(1):26–32. 10.1016/S0003-9993(97)90005-8 9014953

[2] Schlüssel MM, Anjos LA, Kac G. Hand grip strength test and its use in nutritional assessment. Rev Nutr. 2008 4;21(2):233–5. 10.1590/S1415-52732008000200009

[3] Bohannon RW, Magasi S. Identification of dynapenia in older adults through the use of grip strength t-scores. Muscle Nerve. 2015 1;51(1):102–5. 10.1002/mus.24264 24729356 PMC4194263

[4] Bragagnolo R, Caporossi FS, Dock-Nascimento DB, Aguilar-Nascimento JE. Handgrip strength and adductor pollicis muscle thickness as predictors of postoperative complications after major operations of the gastrointestinal tract. E Spen Eur E J Clin Nutr Metab. 2011 2 1;6(1):e21–6.

[5] Costa TB, Neri AL. Indicators of physical activity and frailty in the elderly: data from the FIBRA study in Campinas, São Paulo State, Brazil. Cad Saude Publica. 2011 8;27(8):1537–50. 10.1590/S0102-311X2011000800009 21877002

[6] Bohannon RW. Are hand-grip and knee extension strength reflective of a common construct? Percept Mot Skills. 2012 4;114(2):514–8. 10.2466/03.26.PMS.114.2.514-518 22755456

[7] Bohannon RW. Hand-grip dynamometry predicts future outcomes in aging adults. J Geriatr Phys Ther. 2008;31(1):3–10. 18489802 10.1519/00139143-200831010-00002

[8] Budziareck MB, Duarte RRP, Barbosa-Silva MCG. Reference values and determinants for handgrip strength in healthy subjects. Clin Nutr. 2008 6;27(3):357–62. 10.1016/j.clnu.2008.03.008 18455840

[9] Massy-Westropp NM, Gill TK, Taylor AW, Bohannon RW, Hill CL. Hand grip strength: age and gender stratified normative data in a population-based study. BMC Res Notes. 2011 4 14;4(1):127 10.1186/1756-0500-4-127 21492469 PMC3101655

[10] Anakwe RE, Huntley JS, Mceachan JE. Grip strength and forearm circumference in a healthy population. J Hand Surg Eur Vol. 2007 1 4;32(2):203–9. 10.1016/J.JHSB.2006.11.003 17197064

[11] Chandrasekaran B, Ghosh A, Prasad C, Krishnan K, Chandrasharma B. Age and anthropometric traits predict handgrip strength in healthy normals. J Hand Microsurg. 2010 12;2(2):58–61. 10.1007/s12593-010-0015-6 22282669 PMC3122705

[12] Amaral CA, Portela MC, Muniz PT, Farias ES, Araújo TS, Souza OF. Association of handgrip strength with self-reported diseases in adults in Rio Branco, Acre State, Brazil: a population-based study. Cad Saude Publica. 2015 6;31(6):1313–25. 26200378 10.1590/0102-311X00062214

[13] Flood A, Chung A, Parker H, Kearns V, O’Sullivan TA. The use of hand grip strength as a predictor of nutrition status in hospital patients. Clin Nutr. 2014 2;33(1):106–14. 10.1016/j.clnu.2013.03.003 23615623

[14] Guerra RS, Amaral TF, Sousa AS, Pichel F, Restivo MT, Ferreira S, et al Handgrip strength measurement as a predictor of hospitalization costs. Eur J Clin Nutr. 2015 2;69(2):187–92. 10.1038/ejcn.2014.242 25369830

[15] Guerra RS, Fonseca I, Pichel F, Restivo MT, Amaral TF. Handgrip strength cutoff values for undernutrition screening at hospital admission. Eur J Clin Nutr. 2014 12;68(12):1315–21. 10.1038/ejcn.2014.226 25351643

[16] Mathiowetz V, Kashman N, Volland G, Weber K, Dowe M, Rogers S. Grip and pinch strength: normative data for adults. Arch Phys Med Rehabil. 1985 2;66(2):69–74. 3970660

[17] Fernandes AA, Marins JCB. Teste de força de preensão manual: análise metodológica e dados normativos em atletas. Fisioter Mov. 2011 9;24(3):567–78. 10.1590/S0103-51502011000300021

[18] Josty IC, Tyler MP, Shewell PC, Roberts AH. Grip and pinch strength variations in different types of workers. J Hand Surg Br. 1997 4;22(2):266–9. 9150004 10.1016/s0266-7681(97)80079-4

[19] Kenny RA, Coen RF, Frewen J, Donoghue OA, Cronin H, Savva GM. Normative values of cognitive and physical function in older adults: findings from the Irish Longitudinal Study on Ageing. J Am Geriatr Soc. 2013 5 1;61(s2):S279–90. 10.1111/jgs.12195 23662720

[20] Dodds RM, Syddall HE, Cooper R, Kuh D, Cooper C, Sayer AA. Global variation in grip strength: a systematic review and meta-analysis of normative data. Age Ageing. 2016 3;45(2):209–16. 10.1093/ageing/afv192 26790455 PMC4776623

[21] Dodds RM, Syddall HE, Cooper R, Benzeval M, Deary IJ, Dennison EM, et al Grip strength across the life course: normative data from twelve British studies. PLoS ONE. 2014 12 4;9(12):e113637 10.1371/journal.pone.0113637 25474696 PMC4256164

[22] Dias JA, Ovando AC, Külkamp W, Junior B, Gomes N. Hand grip strength: evaluation methods and factors influencing this measure. Rev Bras Cineantropom Desempenho Hum. 2010 6;12(3):209–16. 10.5007/1980-0037.2010v12n3p209

[23] Roberts HC, Denison HJ, Martin HJ, Patel HP, Syddall H, Cooper C, et al A review of the measurement of grip strength in clinical and epidemiological studies: towards a standardised approach. Age Ageing. 2011 1 7;40(4):423–9. 10.1093/ageing/afr051 21624928

[24] Schlüssel MM, Anjos LA, Vasconcellos MTL, Kac G. Reference values of handgrip dynamometry of healthy adults: a population-based study. Clin Nutr. 2008 8;27(4):601–7. 10.1016/j.clnu.2008.04.004 18547686

[25] Caporrino FA, Faloppa F, Santos JBG, Réssio C, Soares FH do C, Nakachima LR, et al Estudo populacional da força de preensão palmar com dinamômetro Jamar. Rev Bras Ortop. 1998 2;33(2):150–4.

[26] Cueto-Manzano AM, Cortés-Sanabria L, Martínez-Ramírez HR, Rojas-Campos E, Gómez-Navarro B, Castillero-Manzano M. Prevalence of chronic kidney disease in an adult population. Arch Med Res. 2014 8;45(6):507–13. 10.1016/j.arcmed.2014.06.007 24992221

[27] Stevens LA, Li S, Wang C, Huang C, Becker BN, Bomback AS, et al Prevalence of CKD and comorbid illness in elderly patients in the United States: Results from the Kidney Early Evaluation Program (KEEP). Am J Kidney Dis. 2010 3;55(3 Suppl 2):S23–33. 10.1053/j.ajkd.2009.09.035 20172445 PMC4574484

[28] Cochran WG. Sampling techniques. 3rd ed New York: John Wiley & Sons; 1977 428 p.

[29] Silva PLN. Calibration estimation: when and why, how much and how [Internet]. Rio de Janeiro: IBGE; 2004. 35 p. (Textos para discussão. Diretoria de Pesquisas). https://biblioteca.ibge.gov.br/visualizacao/livros/liv66414.pdf

[30] Madeira JL, Simões CCS. Estimativas preliminares da população urbana e rural segundo as unidades da federação, de 1960/1980 por uma nova metodologia. Rev Bras Estat. 1972 3;33(129):3–11.

[31] Amaral TLM, Amaral CA, Portela MC, Monteiro GTR, Vasconcellos MTL. Study of Chronic Diseases (Edoc): methodological aspects. Rev Saude Publica. 2019 1;53(1). 10.11606/S1518-8787.2019053000847 PMC6390664 30726489

[32] American College of Sports Medicine. Manual do ACSM para avaliação da aptidão física relacionada à saúde. Rio de Janeiro: Guanabara Koogan; 2006.

[33] Fess EE. Documentation: essential elements of an upper extremity assessment battery In: Rehabilitation of the hand and upper extremity. 5th ed St Louis: Mosby; 2002 p. 263–84.

[34] Spruit MA, Sillen MJH, Groenen MTJ, Wouters EFM, Franssen FME. New normative values for handgrip strength: results from the UK Biobank. J Am Med Dir Assoc. 2013 10 1;14(10):775.e5–775.e11. 10.1016/j.jamda.2013.06.013 23958225

[35] Steiber N. Strong or weak handgrip? Normative reference values for the German population across the life course stratified by sex, age, and body height. PLoS ONE. 2016 10 4;11(10):e0163917 10.1371/journal.pone.0163917 27701433 PMC5049850

[36] Nahhas RW, Choh AC, Lee M, Chumlea WMC, Duren DL, Siervogel RM, et al Bayesian longitudinal plateau model of adult grip strength. Am J Hum Biol. 2010 10;22(5):648–56. 10.1002/ajhb.21057 20737612 PMC3988672

[37] Oksuzyan A, Maier H, McGue M, Vaupel JW, Christensen K. Sex differences in the level and rate of change of physical function and grip strength in the Danish 1905-cohort study. J Aging Health. 2010 8;22(5):589–610. 10.1177/0898264310366752 20453156 PMC2952838

[38] Taekema DG, Ling CHY, Blauw GJ, Meskers CG, Westendorp RGJ, de Craen AJM, et al Circulating levels of IGF1 are associated with muscle strength in middle-aged- and oldest-old women. Eur J Endocrinol. 2011 1 2;164(2):189–96. 10.1530/EJE-10-0703 21135066

[39] Peoples J, Bailey G. Humanity: An Introduction to Cultural Anthropology. 9th ed USA: Cengage Learning; 2011 498 p.

[40] Arvandi M, Strasser B, Meisinger C, Volaklis K, Gothe RM, Siebert U, et al Gender differences in the association between grip strength and mortality in older adults: results from the KORA-age study. BMC Geriatr. 2016;16(201). 10.1186/s12877-016-0381-4 27903239 PMC5131446

[41] Timpka S, Petersson IF, Zhou C, Englund M. Muscle strength in adolescent men and risk of cardiovascular disease events and mortality in middle age: a prospective cohort study. BMC Med. 2014 4 14;12:62 10.1186/1741-7015-12-62 24731728 PMC4006633

[42] Meller F de O, Ciochetto CR, dos Santos LP, Duval PA, Vieira M de FA, Schäfer AA, et al Association between waist circumference and body mass index of Brazilian women. Cienc Saude Colet. 2014 1;19(1):75–82. 10.1590/1413-81232014191.2000 24473605

[43] Mendes J, Amaral TF, Borges N, Santos A, Padrão P, Moreira P, et al Handgrip strength values of Portuguese older adults: a population based study. BMC Geriatr. 2017 8 23;17(191). 10.1186/s12877-017-0590-5 28835211 PMC5569490

[44] Norman K, Stobäus N, Gonzalez MC, Schulzke J-D, Pirlich M. Hand grip strength: Outcome predictor and marker of nutritional status. Clin Nutr. 2011 4 1;30(2):135–42. 10.1016/j.clnu.2010.09.010 21035927

[45] Leong DP, Teo KK, Rangarajan S, Kutty VR, Lanas F, Hui C, et al Reference ranges of handgrip strength from 125,462 healthy adults in 21 countries: a prospective urban rural epidemiologic (PURE) study. J Cachexia Sarcopenia Muscle. 2016 12;7(5):535–46. 10.1002/jcsm.12112 27104109 PMC4833755

[46] Sternäng O, Reynolds CA, Finkel D, Ernsth-Bravell M, Pedersen NL, Aslan AKD. Factors associated with grip strength decline in older adults. Age Ageing. 2015 1 3;44(2):269–74. 10.1093/ageing/afu170 25362503 PMC4400526

[47] Barrea L, Muscogiuri G, Di Somma C, Tramontano G, De Luca V, Illario M, et al Association between Mediterranean diet and hand grip strength in older adult women. Clin Nutr. 2018 4 3; 10.1016/j.clnu.2018.03.012 29643004

[48] Cruz-Jentoft AJ, Baeyens JP, Bauer JM, Boirie Y, Cederholm T, Landi F, et al Sarcopenia: European consensus on definition and diagnosis: Report of the European Working Group on Sarcopenia in Older People. Age Ageing. 2010 7 1;39(4):412–23. 10.1093/ageing/afq034 20392703 PMC2886201

